# Nurse-Led Ambient AI Scribe for Patient Safety Incident Investigation Reports (Project NARRATE): Retrospective Pre-Post Comparative Document-Quality Study

**DOI:** 10.2196/100775

**Published:** 2026-08-10

**Authors:** Kai Yunn Teo, Liwen Huang, Kelly Chai Yuen Woh, Shin Yuh Ang, Jade Gaik Nai Ng

**Affiliations:** 1 Department of Nursing Administration, Singapore General Hospital, Outram Road, Singapore, Singapore, 169608, Singapore, 65 63265843

**Keywords:** artificial intelligence, ambient AI scribe, nursing, patient safety, incident reporting, documentation quality, learning health system

## Abstract

**Background:**

Patient safety investigation reports support organizational learning only when they are complete, usable, and sufficiently detailed. Conventional free-text reports are often inconsistent and may omit information needed for review and learning. Project NARRATE (Nursing AI-Refined for Accurate Transcription of Events) is a nursing-led ambient artificial intelligence workflow that uses prompts aligned with the World Health Organization Minimal Information Model for Patient Safety Incident Reporting and Learning Systems, Situation-Background-Assessment-Recommendation output, and visible missing-information cues to support structured supervisor reporting.

**Objective:**

This study aimed to compare the completeness and narrative quality of conventional and NARRATE-period supervisor investigation reports for falls and medication administration-related incidents.

**Methods:**

We conducted a retrospective pre-post document-quality study at a tertiary academic medical center in Singapore. We reviewed 150 deidentified completed supervisor investigation reports: 75 conventional reports from June to August 2025 and 75 confirmed NARRATE reports from January to March 2026. NARRATE use was voluntary, and recorded use represented approximately 40% of eligible postimplementation reports. Two blinded reviewers rated reports using a World Health Organization (WHO)–aligned completeness checklist and an adapted 8-domain Physician Documentation Quality Instrument (PDQI). Report-level comparisons were adjusted for repeated reports by the same supervisor using random-intercept linear mixed-effects models. A stratified 60-report plain-paragraph rerating examined whether visible structure influenced ratings.

**Results:**

All 150 reports were analyzed. Unadjusted mean WHO total completeness was 11.81 (SD 3.39) for conventional reports and 13.61 (SD 2.54) for NARRATE reports; the unadjusted difference was 1.80 points, and the cluster-adjusted mean difference was 1.95 (95% CI 0.91‐3.00; *P*<.001). The adapted PDQI mean was 3.61 (SD 0.52) and 4.13 (SD 0.34), respectively; the unadjusted difference was 0.52 points, and the cluster-adjusted mean difference was 0.53 (95% CI 0.37‐0.69; *P*<.001). In the plain-paragraph sensitivity analysis, the completeness advantage remained (adjusted mean difference 1.70, 95% CI 0.27‐3.14; *P*=.02), as did the adapted PDQI mean advantage (adjusted mean difference 0.25, 95% CI 0.06‐0.43; *P*=.009). Explanation, organization, and comprehensibility remained significantly higher after deformatting; actions were borderline (*P*=.05), and synthesis, internal consistency, and fairness/balance were not statistically significant.

**Conclusions:**

Among voluntary early adopters, NARRATE use was associated with more complete reports and higher adapted PDQI mean scores after accounting for supervisor clustering. Because recorded use represented approximately 40% of eligible postimplementation reports and users self-selected, findings may reflect adopter and supervisor characteristics. Results support the structured workflow as a whole, not any single AI component, and do not demonstrate downstream patient-safety effects. Confirmatory evaluation under broader adoption with a concurrent, reliably classified comparison group is needed.

## Introduction

### Background

Patient safety incident reporting systems support learning only when reports contain enough structured, interpretable information to guide investigation and improvement [1-3]. The World Health Organization (WHO) Minimal Information Model for Patient Safety Incident Reporting and Learning Systems (MIM PS) specifies the elements needed for this purpose, including what happened, why it happened, and what was done in response [2,3].

In practice, many incident reports fall short. Prior studies have found incomplete capture of causal factors, latent conditions, patient impact, and follow-up actions, limiting both case review and system-level learning [4,5]. These shortcomings are not merely documentation defects; they weaken the ability of reporting systems to generate meaningful analysis and improvement [4,5].

Reporting alone therefore does not guarantee learning [6-8]. Interventions that improve the completeness, structure, and usability of investigation narratives may strengthen the value of incident reporting for review and action.

### Rationale for the Present Intervention

Ambient AI documentation tools offer one possible response. These scribes can convert spoken narrative into structured draft notes and may reduce documentation burden while improving consistency. Early evaluations of the in-house SingHealth ambient scribe program suggest acceptable note quality and reduced documentation time in outpatient care [9,10]. Whether similar tools improve nursing incident investigation reports remains unclear. To our knowledge, no prior peer-reviewed study has evaluated an ambient AI scribe explicitly aligned with the advanced WHO MIM PS for this purpose.

### Study Context

Project NARRATE (Nursing AI-Refined for Accurate Transcription of Events) was developed to improve the quality of completed nursing incident-investigation reports. Built on SingHealth Note Buddy, it prompts supervisors to narrate key WHO MIM PS elements and generates a Situation-Background-Assessment-Recommendation (SBAR)–structured draft. When a safety-critical element is missing, the draft retains a visible missing-information cue for supervisor completion before final submission. The workflow was intended to support more complete, organized, and review-ready documentation while reducing manual reformatting.

We focused on completed supervisor investigation reports rather than initial frontline incident narratives because they contain the postinvestigation information most relevant to learning, including contributing factors, findings, and proposed actions [2,11].

### Study Objectives and Hypothesis

This study compared the completeness and narrative quality of completed nursing supervisor investigation reports before and after implementation of the NARRATE workflow. We hypothesized that NARRATE-period reports would score higher on WHO MIM PS–aligned completeness and on an adapted 8-domain Physician Documentation Quality Instrument (PDQI)–based narrative-quality assessment [12]. The primary objective was WHO total completeness; the secondary objective was the adapted PDQI mean; additional objectives included domain-specific and interrater reliability analyses.

## Methods

### Study Design

We conducted a retrospective pre-post comparative document-quality study, supplemented by a format-blinded plain-text rerating sensitivity analysis in a stratified subsample to assess whether recognizable SBAR structure influenced ratings. The unit of analysis was the completed supervisor investigation report for a nursing-originated patient safety incident. The exposure was documentation method and period: conventional manual documentation before implementation versus NARRATE-supported documentation after implementation. The primary outcome was WHO total completeness, and the secondary outcome was the adapted PDQI mean. We report the study using the SQUIRE (Standards for Quality Improvement Reporting Excellence) 2.0, supplemented by relevant STROBE (Strengthening the Reporting of Observational Studies in Epidemiology) elements.

### Setting

The study was conducted at Singapore General Hospital, a tertiary academic medical center in Singapore, within a nursing-led patient safety documentation improvement initiative.

### Intervention: NARRATE Supervisor Workflow

NARRATE stands for Nursing AI-Refined for Accurate Transcription of Events. The intervention used SingHealth Note Buddy, an in-house ambient AI scribe operating within approved institutional digital infrastructure. Note Buddy combines automatic speech recognition with a generative large language model, processes audio in real time, and does not retain audio recordings [10]. Users review and edit draft notes before manually transferring verified content into downstream systems [10].

For Project NARRATE, the team configured a supervisor prompt template aligned with the advanced WHO MIM PS. Supervisors narrated incident context, sequence, outcome, causes, contributing and mitigating factors, and actions; the system generated an SBAR-structured draft [13]. When key elements were missing, the draft retained a visible missing-information placeholder. Supervisors were required to verify, edit, and complete the draft before transfer into the incident reporting system. The workflow treated AI output as documentation support rather than an autonomous investigation conclusion.

### AI Governance and Safety Checks

During the study period, NARRATE operated as a stable, postlaunch workflow. The supervisor prompt template was fixed before the study and unchanged throughout, and the underlying ambient-scribe model did not change during the January to March 2026 window. The underlying platform maintained system-level audit logs of user access and draft-generation activity; these platform logs were not analyzed for this study. Every draft required mandatory supervisor verification and editing, including correction of transcription errors and resolution of all missing-information cues, before transfer into the institutional incident-reporting system.

**Figure 1.**
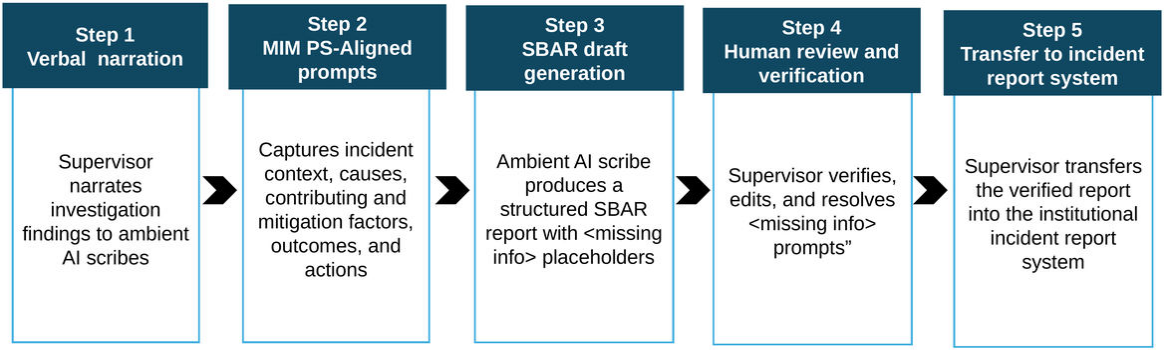
Project NARRATE (Nursing AI-Refined for Accurate Transcription of Events) AI-assisted supervisor investigation documentation workflow. The workflow moves from verbal narration to World Health Organization Minimal Information Model for Patient Safety Incident Reporting and Learning Systems (MIM PS)–aligned prompts, AI-generated Situation-Background-Assessment-Recommendation (SBAR) draft output, human verification, and transfer to the institutional incident reporting system. Supervisors verify all AI-generated content and resolve any missing-information placeholders before final submission.

**Figure 2.**
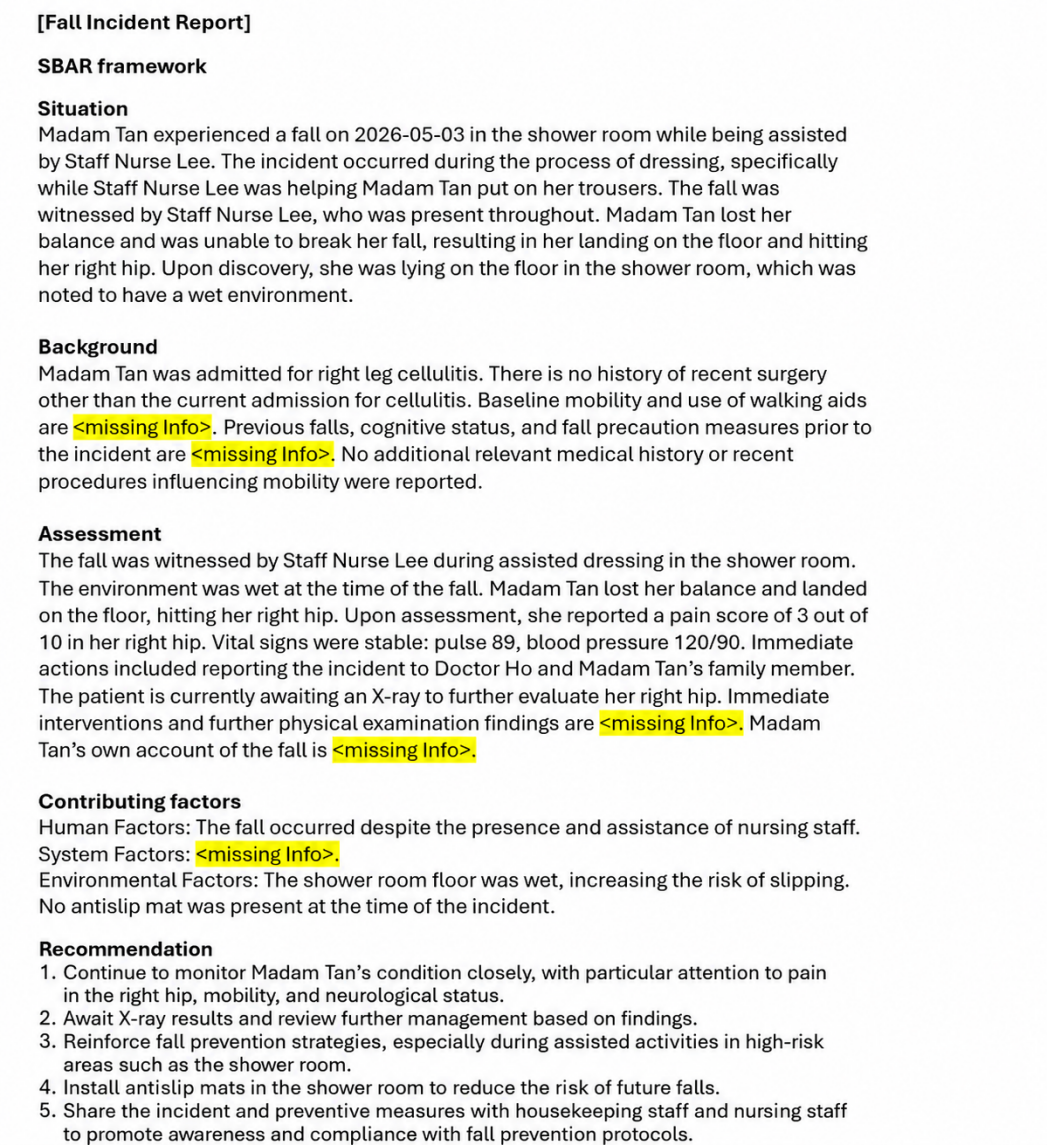
Illustrative sample of NARRATE (Nursing AI-Refined for Accurate Transcription of Events)-generated Situation-Background-Assessment-Recommendation (SBAR) output with missing-information placeholders. This example, based on a fictitious fall incident, shows how the AI-generated draft note retains explicit missing-information markers for safety-critical elements requiring supervisor verification. Supervisors are expected to review and address each placeholder before transferring the verified report into the institutional incident-reporting system. All patient and unit details are fictitious.

Figure 1 illustrates the supervisor workflow from verbal narration through human verification to final report submission. Figure 2 provides an illustrative example of NARRATE-generated SBAR output with missing-information placeholders.

### Comparator: Conventional Documentation

In the conventional process, supervisors documented investigation findings manually in free text after reviewing the incident and gathering information from relevant staff or records. Conventional documentation did not include embedded voice-to-text prompts, SBAR-structured AI output, or visible missing-information cues.

### Sample, Eligibility, and Selection

We analyzed 150 deidentified completed supervisor investigation reports: 75 conventional reports from June to August 2025 and 75 NARRATE-period reports from January to March 2026. Eligible reports were nursing-originated investigation reports with sufficient narrative text to support scoring. The study focused on falls and medication administration-related incidents.

We excluded duplicates, administrative entries without substantive investigation narrative, and reports outside the study windows. NARRATE operated through the institutional ambient-scribe platform, which is technically separate from the incident-reporting system, and its use could not be mandated within the existing reporting workflow. Adoption during the postimplementation period was therefore voluntary (opt-in), and recorded NARRATE use represented approximately 40% of eligible reports. Supervisors who used NARRATE recorded the corresponding report number in a brief operational record maintained for a separate reporting-time initiative rather than for the present analysis. Entries in this record allowed confirmed NARRATE-generated reports to be identified, but the record was not designed as an exhaustive NARRATE-use registry. Absence from the record therefore could not confirm nonuse. We did not construct a postimplementation non-NARRATE comparison group because a complement-defined group could have contained unrecorded NARRATE reports and introduced exposure misclassification.

From the confirmed NARRATE-documented reports, we drew 75 at random, and we drew 75 conventional reports at random from eligible preimplementation reports. Random selection was applied within each pool to avoid investigator selection of individual reports, but it cannot remove adopter self-selection. Early adopters may differ from nonadopters in documentation practice or motivation. We therefore interpret the comparison as the NARRATE workflow as delivered under real-world voluntary uptake rather than under randomized assignment.

All 150 selected reports were analyzable, with no missing report-level or item-level data.

Sample size was not determined a priori; it was fixed by the number of eligible reports available within the institutional study windows. Accordingly, we frame this power calculation as a minimum detectable effect analysis for a fixed sample rather than as a sample-size justification. Using G*Power 3.1, a sample of 150 reports (75 per group) was sufficient to detect a standardized mean difference of *d*=0.46 with approximately 80% power at a 2-sided α of .05 [14]; both primary outcomes exceeded this minimum detectable effect. Domain-level and subgroup analyses were exploratory.

### Outcomes and Measurement

#### Completeness Checklist Development and Content Validity

We assessed the primary outcome, report completeness, using a graded checklist derived from WHO technical guidance and aligned with the advanced WHO MIM PS [2,3]. The checklist grouped items into description (what happened and patient impact), explanation (suspected causes, contributing factors, and mitigating factors), and actions (immediate, corrective, and proposed follow-up actions).

Each item was rated 0=“absent,” 1=“partial or insufficient,” 2=“clear and sufficient for review and learning,” or 9=“not applicable.” Not-applicable items were excluded from the relevant denominator. We calculated WHO total completeness from applicable items and calculated domain subscores as the mean of items within each domain. The full rubric is provided in Multimedia Appendix 1.

Content validity was established through structured review by 3 experts with operational responsibility in patient safety incident review: a Nursing Division patient safety officer, an Office of Patient Safety and Quality manager, and a senior principal pharmacist with medication safety expertise.

Across 2 rounds, panelists evaluated item relevance and contextual appropriateness, and items were refined between rounds. After the second round, all retained items achieved an item-level content validity index of 1.00 [15,16]. The checklist was finalized after the NARRATE prompt template had been locked; the resulting intervention-outcome alignment is addressed in the *Limitations*.

#### Adapted 8-Domain PDQI-Based Narrative Quality Assessment

We assessed narrative quality using an adapted PDQI approach derived from the PDQI [12]. For this incident-investigation context, 8 domains were scored: thoroughness, usefulness, organization, comprehensibility, succinctness, synthesis, internal consistency, and fairness/balance. Each domain was rated from 1 to 5, and the adapted PDQI mean was calculated as the average of the 8 domain scores. Accuracy was excluded a priori because the ambient AI workflow did not retain an audio record or other source transcript for independent audit, so reviewers could not compare the final report with the original verbal narration or otherwise verify factual fidelity beyond the report text itself. This exclusion matters because ambient AI outputs can contain transcription errors that may propagate into the final note, occasionally with potential for harm [17]; reviewers therefore judged the quality of the final written narrative, not the truth of the underlying investigation. NARRATE drafts were always reviewed and edited by a supervisor before submission. The full rubric is provided in Multimedia Appendix 2.

#### Reviewer Training, Blinding, and Scoring

Two reviewers from the Nursing Safety and Quality team independently assessed all 150 deidentified reports. Neither reviewer was involved in NARRATE design, prompt authoring, user training, or implementation. Reports were deidentified and masked by an independent data handler. Direct identifiers were removed from the reviewer-facing reports, while a pseudonymous supervisor code was retained separately for clustered analysis. The 2 reviewers had no role in data preparation or access to the linkage information.

Before formal scoring, the reviewers received written rubric guidance and completed iterative calibration on 10 reports outside the study sample; this process also informed refinement of the rubrics in Multimedia Appendices 1 and 2. All calibration-informed rubric refinement, including finalization of the checklist and its item-level content validity index, was completed before any of the 150 analytic reports were rated, so postcalibration rubric drift did not affect the rated sample.

Residual unblinding remained possible because NARRATE-period reports could retain recognizable SBAR structure. This risk was addressed in the plain-text sensitivity analysis and discussed in the *Limitations*. For the primary analysis, each report was scored independently by both reviewers, and composite scores were calculated as the mean of the 2 ratings after interrater reliability assessment. Individual discrepancies were not adjudicated before the main analysis so that ICC estimates reflected independent ratings.

### Statistical Analysis

We prespecified comparisons of preimplementation versus postimplementation reports on the primary outcome (WHO total completeness) and secondary outcome (adapted PDQI [8-domain] mean), with domain-level comparisons treated as exploratory. We reported means and SDs for continuous scores and frequencies with percentages for categorical characteristics.

Interrater reliability for composite scores was assessed using 2-way random-effects, absolute-agreement intraclass correlation coefficients (ICCs), following the framework described by McGraw and Wong [18]. Single-measure ICCs represented the reliability of 1 reviewer’s score, and average-measure ICCs represented the reliability of the mean across the 2 reviewers.

To account for supervisors contributing multiple reports, all report-level comparisons were refitted using linear mixed-effects models with documentation method as a fixed effect and a random intercept for the documenting supervisor. Models used restricted maximum likelihood estimation; 95% CIs and 2-sided *P* values used Wald normal-theory inference. Supervisors were nested within documentation method because none contributed reports in both study periods. Adjusted mean differences are reported as NARRATE minus conventional. Unadjusted group means, SDs, and Cohen *d* values calculated using the pooled SD were retained as descriptive standardized effect-size summaries, with approximate 95% CIs derived from the independent-groups variance estimator [19,20].

Because Levene tests had indicated unequal variance for the 2 headline outcomes, exchangeable generalized estimating equations with robust SEs were also fitted as robustness checks; conclusions were unchanged. Statistical significance was evaluated at a 2-sided α of .05. We performed 11 prespecified exploratory domain comparisons in the full-sample analysis (3 WHO subscores and 8 adapted PDQI domains). These comparisons were not adjusted for multiplicity, so the family-wise risk of type I error is increased, and domain-level findings should be interpreted as hypothesis-generating. Original reliability and descriptive analyses were conducted using IBM SPSS Statistics for Windows (version 26.0; IBM Corp); the clustered reanalysis was conducted in Python 3.12 using statsmodels 0.14.6.

### Plain-Text Rerating Sensitivity Analysis

Because recognizable SBAR structure could partially unblind reviewers, we conducted a format-blinded sensitivity analysis on a stratified subsample of 60 reports (30 conventional and 30 NARRATE, balanced by incident type) converted to a uniform plain-paragraph format. SBAR labels, section headers, horizontal rules, and other structural cues were removed while preserving report content; an automated audit confirmed that no residual structural markers remained. The rerating used the same 2 reviewers as the main analysis. To prevent reviewers from linking reports to their original SBAR-formatted versions, the data handler (who did not participate in rating) prepared the deformatted set, removed all structural cues, and renumbered and reordered the reports before rerating. Both reviewers were blinded to documentation method and independently rescored all 60 reports using the same WHO completeness checklist and adapted PDQI (8-domain) rubric. Periods were compared using averaged rater scores and the same random-intercept mixed-model approach, with documenting supervisor as the clustering unit; Cohen *d* values were retained as unadjusted descriptive effect sizes.

### Ethical Considerations

We analyzed deidentified existing incident-report text generated through routine institutional reporting for this retrospective quality and safety evaluation; the analytic dataset contained no patient or staff identifiers. The retrospective analysis was reviewed under the institutional Quality Assurance/Service Improvement pathway and was determined not to require review by the SingHealth Centralized Institutional Review Board (institutional reference SHS-RSH-CIRB-4234); this determination for retrospective analysis of existing deidentified records was endorsed by the Research Office, Singapore General Hospital, on May 8, 2026. Individual consent was not required because the study used deidentified existing records and involved no direct interaction with patients or staff. We reported the project in accordance with the SQUIRE 2.0 guidelines for quality-improvement reporting, supplemented by relevant STROBE elements; a completed SQUIRE 2.0 checklist is provided in Checklist 1.

## Results

### Report Sample

A total of 150 nursing-originated supervisor investigation reports were included: 75 conventional and 75 NARRATE reports, covering falls and medication administration-related incidents across both study periods. No cases were excluded. The operational record and associated discussion history identified the documenting supervisor for each selected report. The 75 conventional reports were contributed by 54 supervisors: 39 contributed 1 report, 10 contributed 2, 4 contributed 3, and 1 contributed 4 reports. The 75 NARRATE reports were contributed by 61 supervisors: 49 contributed 1 report, 10 contributed 2, and 2 contributed 3 reports. No supervisor contributed reports in both periods. Sample characteristics by documentation method are summarized in Table 1. The 2 groups were comparable in report length (conventional mean 777, SD 475 words; NARRATE mean 733, SD 254 words; *P*=.49), indicating that NARRATE-period gains were not attributable to longer reports.

**Table 1.** Sample characteristics by documentation method (N=150)^a^.

Characteristic	Conventional (n=75)	NARRATE^b^ (n=75)
Incident type, n (%)		
Fall	41 (55)	45 (60)
Medication administration-related	34 (45)	30 (40)
Sampling period	June-August 2025	January-March 2026
Report length (words), mean (SD)	777 (475)	733 (254)

a Report length was calculated as the word count of the event description. Harm-severity tier, ward type, and reporter seniority were not available in the deidentified dataset. Groups did not differ significantly in report length (*P*=.49).

b NARRATE: Nursing AI-Refined for Accurate Transcription of Events.

### Interrater Reliability

Table 2 summarizes interrater reliability for the composite scores. Agreement was moderate for WHO total completeness and somewhat stronger for the adapted PDQI (8-domain) mean. For WHO total completeness, Cronbach α was 0.829; the single-measure ICC was 0.689 (95% CI 0.577‐0.772; *P*<.001), and the average-measure ICC was 0.816 (95% CI 0.732‐0.872). For the adapted PDQI (8-domain) mean, Cronbach α was 0.853; the single-measure ICC was 0.744 (95% CI 0.664‐0.808; *P*<.001), and the average-measure ICC was 0.854 (95% CI 0.798‐0.894). Across the 9 WHO items (1350 ratings), reviewers agreed exactly on 957 (70.9%) ratings, differed by 1 category on 285 (21.1%), and differed by more than 1 category on 108 (8%). Larger disagreements clustered in the explanation domain, especially contributing factors (38/150, 25.3%), mitigating factors (23/150, 15.3%), and suspected cause (15/150, 10%), whereas description items showed high agreement (5/150, 3.3% differing by >1 category). To confirm that the explanation-domain advantage was not driven by a single reviewer, we examined each reviewer separately: both scored NARRATE-period explanation content higher than conventional content (reviewer 1, mean difference 0.49, *d*=0.81, *P*<.001; reviewer 2, mean difference 0.17, *d*=0.26, *P*=.11). The direction was therefore consistent across reviewers, although the effect was larger and statistically significant for 1 reviewer and smaller and nonsignificant for the other, consistent with the greater interpretive difficulty of explanation items.

**Table 2.** Interrater reliability for composite report-quality scores (N=150).

Outcome	Cronbach α	ICC, single-measure (95% CI)^a^	ICC, average-measure (95% CI)^a^
WHO^b^ total completeness	0.829	0.689 (0.577‐0.772)	0.816 (0.732‐0.872)
Adapted PDQI^c^ (8-domain) mean	0.853	0.744 (0.664‐0.808)	0.854 (0.798‐0.894)

a ICC: intraclass correlation coefficient; ICCs are 2-way random-effects, absolute-agreement. *P*<.001 for all ICCs.

b WHO: World Health Organization.

c PDQI: Physician Documentation Quality Instrument.

### Primary and Secondary Outcomes

NARRATE reports had higher WHO total completeness scores than conventional reports. The observed mean was 13.61 (SD 2.54) for NARRATE reports and 11.81 (SD 3.39) for conventional reports, corresponding to an unadjusted difference of 1.80 points. After accounting for multiple reports contributed by the same supervisor, the cluster-adjusted mean difference was 1.95 points (95% CI 0.91‐3.00; *P*<.001). The corresponding unadjusted descriptive standardized effect size was Cohen *d*=0.60 (95% CI 0.27‐0.93).

NARRATE reports had higher adapted PDQI (8-domain) mean scores. The observed mean was 4.13 (SD 0.34) for NARRATE reports and 3.61 (SD 0.52) for conventional reports, corresponding to an unadjusted difference of 0.52 points. After accounting for multiple reports contributed by the same supervisor, the cluster-adjusted mean difference was 0.53 (95% CI 0.37‐0.69; *P*<.001) points. The corresponding unadjusted descriptive standardized effect size was Cohen *d*=1.18 (95% CI 0.83‐1.53). Exchangeable generalized estimating equation robustness analyses yielded similar estimates for WHO total completeness (1.99, 95% CI 0.94‐3.04; *P*<.001) and adapted PDQI mean (0.53, 95% CI 0.37‐0.70; *P*<.001). Table 3 summarizes group-level results.

**Table 3.** Overall report completeness and narrative quality by documentation method (N=150).

Outcome	Conventional, mean (SD)^a^	NARRATE^b^, mean (SD)^a^	Adjusted mean difference (95% CI)^a^	*P* value^a^	Cohen *d*^c^ (95% CI)
WHO^d^ total completeness	11.81 (3.39)	13.61 (2.54)	1.95 (0.91-3.00)	<.001	0.60 (0.27-0.93)
Adapted PDQI^e^ (8-domain) mean	3.61 (0.52)	4.13 (0.34)	0.53 (0.37-0.69)	<.001	1.18 (0.83-1.53)

a Mean differences, 95% CIs, and *P* values are from restricted maximum likelihood random-intercept linear mixed-effects models with documentation method as a fixed effect and documenting supervisor as a random intercept.

b NARRATE: Nursing AI-Refined for Accurate Transcription of Events.

c Cohen *d* was calculated from unadjusted group means using the pooled SD; 95% CIs for *d* were derived from the independent-groups variance estimator.

d WHO: World Health Organization.

e PDQI: Physician Documentation Quality Instrument.

### Exploratory WHO Completeness Domain Analysis

Exploratory WHO domain analysis suggested that the overall completeness improvement was driven mainly by explanation and actions (Table 4). In cluster-adjusted models, the description subscore increased only slightly from 1.86 (SD 0.32) to 1.94 (SD 0.18; adjusted mean difference 0.08, 95% CI −0.01 to 0.16; *P*=.07; *d*=0.30), suggesting a ceiling effect. Explanation increased from 1.00 (SD 0.58) to 1.33 (SD 0.51; adjusted mean difference 0.34, 95% CI 0.15‐0.52; *P*<.001; *d*=0.60), and actions increased from 1.27 (SD 0.43) to 1.43 (SD 0.31; adjusted mean difference 0.19, 95% CI 0.06‐0.32; *P*=.004; *d*=0.43). Cohen *d* values are unadjusted descriptive effects. These subscore findings should be interpreted as exploratory.

**Table 4.** World Health Organization minimal information model for patient safety incident reporting and learning systems–aligned completeness subscores by documentation method (N=150)^a^.

WHO^b^ domain^c^	Conventional, mean (SD)^d^	NARRATE^e^, mean (SD)^d^	Adjusted mean difference (95% CI)^d^	*P* value^d^	Cohen *d*^f^ (95% CI)
Description	1.86 (0.32)	1.94 (0.18)	0.08 (−0.01 to 0.16)	.07	0.30 (−0.03 to 0.62)
Explanation	1.00 (0.58)	1.33 (0.51)	0.34 (0.15 to 0.52)	<.001	0.60 (0.28 to 0.93)
Actions	1.27 (0.43)	1.43 (0.31)	0.19 (0.06 to 0.32)	.004	0.43 (0.11 to 0.76)

a Subscore values are means of constituent items within each domain.

b WHO: World Health Organization.

c Domain-level analyses were exploratory.

d Mean differences, 95% CIs, and *P* values are from restricted maximum likelihood random-intercept linear mixed-effects models clustered by documenting supervisor.

e NARRATE: Nursing AI-Refined for Accurate Transcription of Events.

f Cohen *d* values are unadjusted descriptive effects. Domain-level analyses were exploratory.

### Exploratory Adapted PDQI (8-Domain) Domain Analysis

Exploratory adapted PDQI (8-domain) analysis showed higher unadjusted scores in the NARRATE group across all 8 measured domains (Table 5). In cluster-adjusted models, differences were statistically significant for thoroughness, usefulness, organization, comprehensibility, synthesis, internal consistency, and fairness/balance. Succinctness improved numerically from 4.31 (SD 0.80) to 4.47 (SD 0.57) but was not statistically significant (adjusted mean difference 0.12, 95% CI −0.12 to 0.37; *P*=.31; *d*=0.23). The largest adjusted absolute increase was in organization, and the largest unadjusted standardized effects were for organization (*d*=2.10), comprehensibility (*d*=1.33), and fairness/balance (*d*=1.32). Fairness/balance nevertheless remained the lowest-rated adapted PDQI domain in both groups.

**Table 5.** Adapted Physician Documentation Quality Instrument (8-domain) narrative quality domain scores by documentation method (N=150).

Adapted PDQI^a^ (8-domain) domain^b^	Conventional, mean (SD)^c^	NARRATE^d^, mean (SD)^c^	Adjusted mean difference (95% CI)^c^	*P* value	Cohen *d*^e^ (95% CI)
Thorough	3.61 (1.08)	4.21 (0.85)	0.66 (0.32 to 1.00)	<.001	0.62 (0.30 to 0.95)
Useful	3.70 (1.09)	4.27 (0.88)	0.61 (0.27 to 0.95)	<.001	0.57 (0.25 to 0.90)
Organized	3.59 (0.52)	4.65 (0.48)	1.04 (0.86 to 1.23)	<.001	2.10 (1.70 to 2.50)
Comprehensible	4.01 (0.49)	4.57 (0.35)	0.56 (0.41 to 0.72)	<.001	1.33 (0.97 to 1.68)
Succinct	4.31 (0.80)	4.47 (0.57)	0.12 (−0.12 to 0.37)	.31	0.23 (−0.09 to 0.55)
Synthesized	3.43 (1.14)	3.96 (0.87)	0.54 (0.20 to 0.88)	.002	0.53 (0.20 to 0.85)
Internal consistency	3.47 (0.42)	3.63 (0.34)	0.17 (0.04 to 0.31)	.01	0.43 (0.11 to 0.76)
Fair/balanced	2.79 (0.39)	3.31 (0.41)	0.54 (0.40 to 0.67)	<.001	1.32 (0.97 to 1.68)

a PDQI: Physician Documentation Quality Instrument.

b Domain-level analyses were exploratory.

c Mean differences, 95% CIs, and *P* values are from restricted maximum likelihood random-intercept linear mixed-effects models clustered by documenting supervisor.

d NARRATE: Nursing AI-Refined for Accurate Transcription of Events.

e Cohen *d* was calculated from unadjusted group means using the pooled SD; 95% CIs were derived from the independent-groups variance estimator.

### Plain-Text Rerating Sensitivity Analysis

After reports were re-rendered into a uniform plain-paragraph format and a stratified subsample was rerated, the completeness advantage remained in the cluster-adjusted analysis (adjusted mean difference 1.70, 95% CI 0.27‐3.14; *P*=.02; unadjusted *d*=0.59, 95% CI 0.08‐1.11), as did the explanation subscore (adjusted mean difference 0.25, 95% CI 0.05‐0.45; *P*=.02; *d*=0.63). The actions subscore still favored NARRATE but was borderline (adjusted mean difference 0.16, 95% CI −0.0007 to 0.32; *P*=.05; *d*=0.50). The adapted PDQI (8-domain) mean remained higher in NARRATE reports but attenuated to a moderate effect (adjusted mean difference 0.25, 95% CI 0.06‐0.43; *P*=.009; *d*=0.64, 95% CI 0.12‐1.16). Among PDQI domains, organization (adjusted mean difference 0.43, 95% CI 0.19‐0.67; *P*<.001; *d*=0.92) and comprehensibility (adjusted mean difference 0.45, 95% CI 0.21‐0.69; *P*<.001; *d*=0.93) remained higher after deformatting, whereas synthesis, internal consistency, and fairness/balance were not statistically significant. These results suggest that the completeness finding and part of the organization and comprehensibility advantage were robust to removal of structural formatting, whereas several other narrative-quality differences were more format-dependent. Cluster-adjusted mean differences, clustered *P* values, and unadjusted effect sizes are summarized in Table 6; corresponding full-sample results are reported in Tables 3-5.

**Table 6.** Format-blinded plain-text sensitivity-analysis results (n=60)^a^.

Outcome	Adjusted mean difference (95% CI)	Cohen *d*^b^ (95% CI)	*P* value^c^
WHO^d^ total completeness	1.70 (0.27 to 3.14)	0.59 (0.08 to 1.11)	.02
Description subscore	0.13 (−0.004 to 0.26)	0.48 (−0.03 to 0.99)	.06
Explanation subscore	0.25 (0.05 to 0.45)	0.63 (0.11 to 1.15)	.02
Actions subscore	0.16 (−0.001 to 0.32)	0.50 (−0.01 to 1.01)	.05
Adapted PDQI^e^ (8-domain) mean	0.25 (0.06 to 0.43)	0.64 (0.12 to 1.16)	.009
Thorough	0.26 (−0.13 to 0.65)	0.30 (−0.21 to 0.81)	.19
Useful	0.26 (−0.13 to 0.65)	0.30 (−0.21 to 0.81)	.19
Organized	0.43 (0.19 to 0.67)	0.92 (0.39 to 1.45)	<.001
Comprehensible	0.45 (0.21 to 0.69)	0.93 (0.40 to 1.46)	<.001
Succinct	0.13 (−0.16 to 0.41)	0.18 (−0.32 to 0.69)	.38
Synthesized	0.21 (−0.03 to 0.45)	0.39 (−0.12 to 0.90)	.09
Internal consistency	0.07 (−0.14 to 0.29)	0.20 (−0.30 to 0.71)	.51
Fair/balanced	0.13 (−0.09 to 0.36)	0.30 (−0.21 to 0.81)	.25

a Positive values favor NARRATE. Corresponding full-sample results are reported in Tables 3–5.

b Cohen *d* was calculated from unadjusted group means using the pooled SD; 95% CIs were derived from the independent-groups variance estimator.

c *P* values are from restricted maximum likelihood random-intercept linear mixed-effects models clustered by documenting supervisor.

d WHO: World Health Organization.

e PDQI: Physician Documentation Quality Instrument.

## Discussion

### Principal Findings

In this retrospective pre-post comparative document-quality study of 150 completed supervisor investigation reports, NARRATE-period reports were more complete and had higher adapted PDQI (8-domain) mean scores than conventional reports. The plain-paragraph sensitivity analysis refined that picture: the completeness advantage and explanation-domain gain persisted after structural formatting was removed, the actions-domain gain attenuated, and organization and comprehensibility remained higher but with smaller effects. By contrast, synthesis, internal consistency, and fairness/balance no longer differed significantly. The most defensible interpretation is therefore improved completeness, explanation content, and some aspects of narrative organization and clarity, with several other narrative-quality differences partly mediated by visible format.

Description was already well documented in conventional reports, which likely limited room for improvement. By contrast, explanation and action content require supervisors to organize findings, identify contributing factors, and specify follow-up, making them more plausible targets for structured prompting [21,22].

### Interpretation of WHO Completeness Findings

The completeness findings support prompt-guided documentation aligned to the advanced WHO MIM PS. NARRATE did not appear merely to lengthen or polish reports; it improved the explanatory and action-oriented elements most relevant to review and learning [2,3].

This matters because reports that omit causes or contributing factors are weaker starting points for investigation and root-cause analysis [2,4,23]. By prompting these elements and making omissions visible through missing-information cues, NARRATE may help turn routine supervisor reports into more usable review documents.

The actions-domain gain should still be interpreted cautiously. The effect was modest in the full sample and only borderline in the plain-text analysis, suggesting improved documentation of actions more than proof of stronger action planning itself. Future iterations could prompt more explicitly for system-level actions, accountability, and timelines.

### Interpretation of Adapted PDQI (8-Domain) Findings

The adapted PDQI findings require domain-specific interpretation rather than a single claim of globally improved narrative quality [12].

In the full sample, organization, comprehensibility, and fairness/balance showed the largest effects, consistent with the visible SBAR format, which may reduce reviewer cognitive load [13,24,25]. The plain-paragraph sensitivity analysis showed that this pattern was only partly format-driven: organization and comprehensibility remained significantly higher after SBAR labels and structural cues were removed, although both attenuated substantially. By contrast, synthesis, internal consistency, and fairness/balance were no longer significant, and the adapted PDQI (8-domain) mean decreased from a large to a moderate effect.

The narrative-quality findings therefore appear to reflect a mixed mechanism. Some gains, particularly in organization and comprehensibility, seem to extend beyond recognition of the formatted template, whereas a substantial portion of the synthesis, internal consistency, and fairness/balance advantage is likely format-mediated. This does not make structure unimportant in practice; structured presentation may still improve readability and navigation even if it should not be interpreted here as an independent content gain.

Succinctness was the only PDQI domain without a significant full-sample gain, which is plausible because SBAR summaries and completion prompts can lengthen reports. Even so, succinctness remained high in the NARRATE period, suggesting that improved completeness did not come at the cost of markedly poorer brevity.

Fairness/balance had the lowest mean PDQI (8-domain) domain score in both groups and became non-significant after deformatting. This suggests that fairness in incident reporting depends less on formatting than on investigation depth, avoidance of blame, and adequate attention to system factors [26,27]. The lower variance of adapted PDQI (8-domain) mean scores in the NARRATE group (SD 0.34 vs 0.52) is also a mixed finding: more standardized documentation may improve consistency, but it may also compress idiosyncratic detail that remains useful for review.

### Interrater Reliability and Measurement Implications

Interrater reliability was somewhat stronger for adapted PDQI (8-domain) mean than for WHO total completeness, but both composites were within an acceptable range for group-level comparisons. Larger WHO disagreements clustered in the explanation items, which require more interpretive judgment than the more factual description items. These findings support continued use of detailed anchors and suggest that future studies could consider adjudication or consensus scoring for borderline causal items.

### Comparison With Prior Work

These findings extend prior concerns that incomplete incident reports limit patient safety learning [4,5]. Rather than evaluating a generic transcription tool, this study examined a workflow that combined voice-to-text capture, WHO MIM PS-aligned prompts, visible omission cues, and SBAR output. The results suggest that prompt-guided narration may improve the capture of learning-relevant elements, although they remain documentation-quality outcomes rather than evidence of reduced harm.

### Implications for Nursing Practice, Leadership, and Safety Governance

For nursing leaders, the practical implication is that AI-enabled documentation may be more valuable as a way to improve the quality of incident-review inputs than as a pure efficiency tool [3,4,10,23,28]. When designed to improve the structure, completeness, and usability of investigation narratives, workflows like NARRATE may help supervisors produce reports that are easier to review and more useful for learning.

Implementation should nevertheless preserve professional accountability. Supervisors must verify AI-generated content, correct transcription errors, resolve missing-information placeholders, and ensure that recommendations are clinically and operationally appropriate. Governance should also address confidentiality, responsible AI use, prompt versioning, audit trails, user training, and equity of use across roles and settings [29]. Because accuracy was not assessed, these findings indicate more complete and better-organized reports, not more factually correct ones. No draft-to-final accuracy audit was performed, and accuracy assurance in this workflow rests on mandatory supervisor verification of every draft. Ambient-scribe transcription errors can propagate to the final note, sometimes with potential for moderate-to-severe harm [17]. Completeness and organization gains therefore do not substitute for accuracy assurance and should not be taken as evidence that AI-generated incident narratives can be relied upon without source-data verification in higher-stakes investigations.

### Strengths

This study has several strengths: a guidance-informed completeness framework aligned to WHO reporting principles, expert content-validity review of the completeness checklist, a 150-report analytic sample, blinded dual-reviewer scoring with quantified interrater reliability, and parallel examination of both total scores and domain-specific patterns.

### Limitations

This study has several limitations. The retrospective pre-post design cannot establish causality, and secular changes cannot be entirely excluded. No additional reporting-quality training or audit-feedback initiative was conducted in the interval, and the institutional incident-reporting system was unchanged; the postimplementation window represented a stable postlaunch period with a fixed prompt template.

NARRATE use was voluntary, and recorded use represented approximately 40% of eligible postimplementation reports. Because the operational record was maintained for another purpose and was not exhaustive, a reliably classified postimplementation non-NARRATE group could not be constructed: absence from the record could not confirm nonuse, and a complement-defined group could have included unrecorded NARRATE reports. The NARRATE group therefore reflects self-selected early adopters, who may differ from nonadopters in motivation, experience, or documentation practice; observed differences may consequently overestimate effects under mandated or universal adoption.

Although the mixed models accounted for correlation among multiple reports from the same supervisor, no supervisor contributed reports in both periods. Documentation method was therefore completely nested within supervisor pool, and clustering adjustment cannot eliminate confounding by different supervisor composition. The comparison cannot fully separate workflow effects from adopter or supervisor characteristics and should be interpreted as performance under real-world voluntary uptake rather than as an unbiased causal contrast. Residual unblinding was possible because NARRATE reports could retain recognizable SBAR structure; the plain-text analysis suggests that some narrative-quality effects were format-sensitive. Because we assessed final supervisor-edited text, we cannot separate the independent effects of transcription, prompting, missing-information cues, and human editing; effects should be attributed to the workflow as a whole.

The prompt template and completeness checklist were both aligned with the advanced WHO MIM PS, creating intervention-outcome alignment. Because the checklist was finalized after the NARRATE prompt template was locked, this criterion-contamination risk is directional: the checklist could in principle have been shaped by what the prompt already produced. To limit this risk, the content-validity panel evaluated items against WHO MIM PS source guidance rather than the specific NARRATE prompt wording, the checklist graded sufficiency rather than simple field completion, and the adapted PDQI findings partly persisted after deformatting. Nonetheless, confirmatory work using a completeness instrument developed independently of the NARRATE prompts is needed.

The adapted PDQI underwent content adaptation but not full construct validation, and the study assessed documentation quality rather than action quality, timeliness, recurrence, factual accuracy, or patient-safety outcomes. No draft-to-final accuracy audit was performed; accuracy assurance rested on mandatory supervisor verification of every draft. Granular case-mix variables were unavailable, and the study was conducted at a single Singapore tertiary center, limiting causal inference and generalizability.

### Future Work

Future work should test whether improved completeness translates into stronger action planning, faster review, better aggregation of contributing factors, and measurable organizational learning. Confirmatory studies should include concurrent, reliably classified nonadopter comparison groups or phased implementation designs that permit within-supervisor comparisons, helping distinguish workflow effects from adopter and supervisor characteristics. Larger multicenter studies could assess implementation fidelity, adoption, and equity across roles and settings. Prospective studies should retain auditable source material so that documentation quality and draft-to-final accuracy can be evaluated together.

### Conclusions

Project NARRATE is a nursing-led application of ambient AI documentation support to incident-investigation reporting. Among voluntary early adopters in this pre-post study, NARRATE-supported reports were associated with greater completeness and higher adapted PDQI (8-domain) mean scores after accounting for repeated reports by supervisors, and completeness gains persisted after structural formatting was removed. The findings do not establish that NARRATE alone caused these differences because adopter self-selection and nonoverlapping supervisor pools remain potential explanations. The strongest evidence supports gains in completeness, explanation content, and selected aspects of narrative organization and clarity, whereas several other narrative-quality differences were format-sensitive. Broader prospective evaluation with concurrent controls is required, with AI-generated documentation remaining subject to human verification and governance.

## Supplementary material

10.2196/100775 Multimedia Appendix 1 Advanced WHO Minimal Information Model for Patient Safety Incident Reporting and Learning Systems–aligned graded completeness checklist, adapted from WHO technical guidance and the MIM PS User Guide [2,3].

10.2196/100775 Multimedia Appendix 2 Eight-domain narrative-quality rubric adapted from the Physician Documentation Quality Instrument-9 described by Stetson et al [12].

10.2196/100775 Checklist 1 SQUIRE 2.0 checklist.

## Abbreviations

ICC: intraclass correlation coefficient
MIM PS: Minimal Information Model for Patient Safety Incident Reporting and Learning Systems
NARRATE: Nursing AI-Refined for Accurate Transcription of Events
PDQI: Physician Documentation Quality Instrument
SBAR: Situation-Background-Assessment-Recommendation
SQUIRE: Standards for Quality Improvement Reporting Excellence
STROBE: Strengthening the Reporting of Observational Studies in Epidemiology
WHO: World Health Organization
