# Supplementary material for: Nurse-Led Ambient AI Scribe for Patient Safety Incident Investigation Reports (Project NARRATE): Retrospective Pre-Post Comparative Document-Quality Study
Source: JMIR Nurs. 2026 Aug 10;9:e100775. doi: 10.2196/100775 (PMC13456676; doi:10.2196/100775)
Supplement: Supplementary file 1 [file nursing-v9-e100775-s001.docx]

Advanced WHO MIM PS-aligned graded completeness checklist

# Overview

The checklist was adapted from the World Health Organization (WHO) Minimal Information Model for Patient Safety Incident Reporting and Learning Systems [1,2] and aligned to narrative elements relevant to completed supervisor investigation reports. It contains 9 items grouped into 3 conceptual domains: Description, Explanation, and Actions.

**Table S1.** Purpose and scope of the completeness checklist.

**Purpose of this appendix**

This checklist was used to assess the completeness of completed nursing supervisor investigation narratives. It focuses on whether key free-text narrative elements were documented, rather than whether fixed administrative metadata fields were completed.

**Table S2.** WHO MIM PS-aligned completeness domains, items, constructs assessed, and score ranges.

| **Domain** | **Items** | **Construct assessed** | **Score range** |
| --- | --- | --- | --- |
| **Description** | Items 1-2 | What happened and what the patient outcome or impact was | 0-4 |
| **Explanation** | Items 3-5 | Why or how the incident occurred, including contributing and mitigating factors | 0-6 |
| **Actions** | Items 6-9 | Immediate, follow-up, proposed, and preventive actions | 0-8 |
| **Total completeness score** | Items 1-9 | Overall completeness of the supervisor investigation narrative | 0-18 |

# General Scoring Scheme

**Table S3.** General scoring scheme for checklist item ratings.

| **Score** | **Interpretation** | **General meaning** |
| --- | --- | --- |
| **2** | Clear / specific / sufficient | The information is explicitly documented and sufficiently specific for review. |
| **1** | Partial / vague / insufficient | The information is present but incomplete, implied, generic, or insufficiently specific. |
| **0** | Absent | The information is not documented in a meaningful way. |
| **9** | Not applicable | Reserved for items that genuinely could not apply to the incident type. In the analytic dataset reported in the manuscript, no report-level summary variables required exclusion because of not applicable scoring. |

# Narrative Elements Assessed

**Table S4.** Overview of checklist items, domains, narrative elements, and assessment focus.

| **Item** | **Domain** | **Narrative element** | **What reviewers looked for** |
| --- | --- | --- | --- |
| **1** | Description | Incident synopsis | Whether the narrative provides a clear account of what happened, including the event itself and enough contextual detail for the incident to be understood without referring to other records. |
| **2** | Description | Outcome or impact | Whether the narrative states the actual or potential effect on the patient, including no harm, near miss, harm, or relevant patient status after the event. |
| **3** | Explanation | Suspected cause or failure mechanism | Whether the narrative explains how or why the incident occurred, beyond simply stating that an incident happened. |
| **4** | Explanation | Contributing factors | Whether the narrative identifies factors that increased the likelihood of the incident. Factors may relate to people, process, equipment or technology, environment, communication, workload, supervision, or other system conditions. |
| **5** | Explanation | Mitigating factors | Whether the narrative documents any factor that reduced harm or prevented a worse outcome, such as early detection, partial defence, immediate assistance, or another protective mechanism. |
| **6** | Actions | Immediate actions taken | Whether the narrative documents what was done at the time of the incident to manage the patient, contain the problem, or escalate the event appropriately. |
| **7** | Actions | Proposed actions aligned with contributing factors | Whether the report documents recommended actions that logically address the contributing factors or failure mechanism identified in the same narrative. |
| **8** | Actions | Follow-up investigation or review steps | Whether the report documents specific follow-up review or investigation activities after the incident, such as staff interviews, chart review, audit, case review, patient/family follow-up, or other investigatory steps. |
| **9** | Actions | Corrective actions to prevent recurrence | Whether the narrative documents a concrete preventive step intended to reduce the likelihood of a similar incident recurring. |

# Item Definitions and Scoring Anchors

## Item 1. Incident synopsis

**Domain:** Description

**What was assessed:** Whether the narrative provides a clear account of what happened, including the event itself and enough contextual detail for the incident to be understood without referring to other records.

**Table S5.** Scoring anchors for Item 1, incident synopsis.

| **Score** | **Anchor label** | **Scoring anchor** |
| --- | --- | --- |
| **2** | Clear / specific / sufficient | The incident is described clearly and specifically, with enough contextual detail to understand the event and its immediate circumstances. Details may include who was involved, what occurred, when and where it occurred, and clinically relevant event details. |
| **1** | Partial / vague / insufficient | The event is mentioned, but the synopsis is incomplete, vague, or missing key contextual detail. |
| **0** | Absent | The incident is not meaningfully described. |

## Item 2. Outcome or impact

**Domain:** Description

**What was assessed:** Whether the narrative states the actual or potential effect on the patient, including no harm, near miss, harm, or relevant patient status after the event.

**Table S6.** Scoring anchors for Item 2, outcome or impact.

| **Score** | **Anchor label** | **Scoring anchor** |
| --- | --- | --- |
| **2** | Clear / specific / sufficient | The outcome or impact is explicitly stated, such as no harm, clinical stability, near miss, or a specific injury or clinical effect. |
| **1** | Partial / vague / insufficient | An outcome is implied but not clearly stated, or only indirect evidence of impact is provided. |
| **0** | Absent | No patient outcome or impact is documented. |

## Item 3. Suspected cause or failure mechanism

**Domain:** Explanation

**What was assessed:** Whether the narrative explains how or why the incident occurred, beyond simply stating that an incident happened.

**Table S7.** Scoring anchors for Item 3, suspected cause or failure mechanism.

| **Score** | **Anchor label** | **Scoring anchor** |
| --- | --- | --- |
| **2** | Clear / specific / sufficient | A specific cause or failure mechanism is described clearly enough for the reader to understand what went wrong in the process, behaviour, or clinical sequence. |
| **1** | Partial / vague / insufficient | A generic cause label is used, but the specific failure mechanism is not described. |
| **0** | Absent | No cause or reason is stated. |

## Item 4. Contributing factors

**Domain:** Explanation

**What was assessed:** Whether the narrative identifies factors that increased the likelihood of the incident. Factors may relate to people, process, equipment or technology, environment, communication, workload, supervision, or other system conditions.

**Table S8.** Scoring anchors for Item 4, contributing factors.

| **Score** | **Anchor label** | **Scoring anchor** |
| --- | --- | --- |
| **2** | Clear / specific / sufficient | At least one clearly identifiable contributing factor is explicitly named and linked to the incident. |
| **1** | Partial / vague / insufficient | A contributing factor is vague, weakly implied, or stated without enough specificity. |
| **0** | Absent | No contributing factor is documented. |

## Item 5. Mitigating factors

**Domain:** Explanation

**What was assessed:** Whether the narrative documents any factor that reduced harm or prevented a worse outcome, such as early detection, partial defence, immediate assistance, or another protective mechanism.

**Table S9.** Scoring anchors for Item 5, mitigating factors.

| **Score** | **Anchor label** | **Scoring anchor** |
| --- | --- | --- |
| **2** | Clear / specific / sufficient | A specific mitigating factor or protective mechanism is documented. |
| **1** | Partial / vague / insufficient | Harm reduction or mitigation is implied, but the protective factor is not clearly specified. |
| **0** | Absent | No mitigating factor is documented. |

## Item 6. Immediate actions taken

**Domain:** Actions

**What was assessed:** Whether the narrative documents what was done at the time of the incident to manage the patient, contain the problem, or escalate the event appropriately.

**Table S10.** Scoring anchors for Item 6, immediate actions taken.

| **Score** | **Anchor label** | **Scoring anchor** |
| --- | --- | --- |
| **2** | Clear / specific / sufficient | Immediate actions are clearly documented and include a sufficiently specific clinical, containment, or escalation response. |
| **1** | Partial / vague / insufficient | Some immediate action is mentioned, but documentation is limited, incomplete, or only partly informative. |
| **0** | Absent | No immediate action is described. |

## Item 7. Proposed actions aligned with contributing factors

**Domain:** Actions

**What was assessed:** Whether the report documents recommended actions that logically address the contributing factors or failure mechanism identified in the same narrative.

**Table S11.** Scoring anchors for Item 7, proposed actions aligned with contributing factors.

| **Score** | **Anchor label** | **Scoring anchor** |
| --- | --- | --- |
| **2** | Clear / specific / sufficient | A proposed action is clearly documented and meaningfully aligned with the identified cause or contributing factor. |
| **1** | Partial / vague / insufficient | A recommendation is present, but it is generic, weakly targeted, or only loosely aligned with the identified issue. |
| **0** | Absent | No proposed action is documented. |

## Item 8. Follow-up investigation or review steps

**Domain:** Actions

**What was assessed:** Whether the report documents specific follow-up review or investigation activities after the incident, such as staff interviews, chart review, audit, case review, patient/family follow-up, or other investigatory steps.

**Table S12.** Scoring anchors for Item 8, follow-up investigation or review steps.

| **Score** | **Anchor label** | **Scoring anchor** |
| --- | --- | --- |
| **2** | Clear / specific / sufficient | At least one specific follow-up investigation or review step is clearly documented. |
| **1** | Partial / vague / insufficient | Follow-up is mentioned only in vague or non-specific terms. |
| **0** | Absent | No follow-up review or investigation step is documented. |

## Item 9. Corrective actions to prevent recurrence

**Domain:** Actions

**What was assessed:** Whether the narrative documents a concrete preventive step intended to reduce the likelihood of a similar incident recurring.

**Table S13.** Scoring anchors for Item 9, corrective actions to prevent recurrence.

| **Score** | **Anchor label** | **Scoring anchor** |
| --- | --- | --- |
| **2** | Clear / specific / sufficient | At least one clear, concrete, and repeatable corrective action to prevent recurrence is documented. |
| **1** | Partial / vague / insufficient | A generic reminder, counselling statement, or thin preventive action is mentioned without a clearly durable or specific preventive mechanism. |
| **0** | Absent | No corrective action to prevent recurrence is documented. |

# Score Calculation

**Table S14.** Score calculation formulas and ranges for checklist totals and domain subscores.

| **Score** | **Formula** | **Possible range** |
| --- | --- | --- |
| **Description domain** | Item 1 + Item 2 | 0-4 |
| **Explanation domain** | Item 3 + Item 4 + Item 5 | 0-6 |
| **Actions domain** | Item 6 + Item 7 + Item 8 + Item 9 | 0-8 |
| **Total completeness score** | Sum of Items 1-9 | 0-18 |
| **Optional domain mean** | Domain sum divided by number of items in that domain | 0-2 |

# Reviewer Scoring Principles

- Rate only what is explicitly documented in the completed report text; do not infer undocumented information from clinical knowledge or assumptions.
- Score the completed narrative as a whole, including multiple dated entries if present. Later entries may increase a score if they add relevant detail.
- Score documentation completeness, not whether the event interpretation is clinically correct.
- If the AI-generated note contains a placeholder such as <missing info>, treat the unresolved placeholder as evidence that the information was not fully documented. Score the relevant item as absent or partial depending on whether any usable detail is present elsewhere in the report.
- When a code of 9 is used, it should be reserved for genuinely non-applicable items and handled consistently during analysis.
