# Supplementary material for: Nurse-Led Ambient AI Scribe for Patient Safety Incident Investigation Reports (Project NARRATE): Retrospective Pre-Post Comparative Document-Quality Study
Source: JMIR Nurs. 2026 Aug 10;9:e100775. doi: 10.2196/100775 (PMC13456676; doi:10.2196/100775)
Supplement: Supplementary file 2 [file nursing-v9-e100775-s002.docx]

Multimedia Appendix 2. Adapted 8-Domain PDQI–Based Narrative Quality Rubric

# Overview

The eight-domain narrative-quality rubric was adapted from the Physician Documentation Quality Instrument-9 (PDQI-9) described by Stetson et al [12], with wording revised for patient safety incident investigation narratives rather than physician progress notes. The rubric evaluates the quality of the final written narrative, not the factual accuracy of the investigation against external source data.

**Table S1.** Purpose and scope of the adapted PDQI-based narrative-quality rubric.

**Purpose of this appendix**

This rubric was used to assess the narrative quality of completed nursing supervisor investigation reports. It complements the WHO MIM PS-aligned completeness checklist [3] by assessing clarity, organization, usefulness, synthesis, and balance of the written narrative.

**Table S2.** Important adaptation for this study: exclusion of the accuracy domain.

Important adaptation for this study The original PDQI accuracy domain was not scored because factual accuracy cannot be determined from final report text alone without independent source data, such as audio recordings, investigation notes, or chart review. The adapted score therefore reflects 8-domain narrative quality and usability, not independent factual verification.

**Table S3.** General scoring scheme for adapted PDQI ratings.

| **Score** | **Interpretation** | **General meaning** |
| --- | --- | --- |
| **5** | Extremely | The criterion is strongly demonstrated throughout the narrative. |
| **4** | Very | The criterion is demonstrated well, with only minor limitations. |
| **3** | Moderately | The criterion is partly demonstrated, but there are clear opportunities for improvement. |
| **2** | Slightly | The criterion is weakly demonstrated and substantially limited. |
| **1** | Not at all | The criterion is absent or poorly demonstrated. |

All adapted PDQI domains were scored on a 5-point Likert-type scale. Scores of 2 and 4 were used when the narrative fell between the adjacent descriptive levels.

# Adapted Narrative Quality Domains

**Table S4.** Adapted PDQI domains and constructs assessed.

| **Item** | **Domain** | **Construct assessed** |
| --- | --- | --- |
| **1** | Thorough | Assesses whether the narrative contains enough relevant information to support review of the incident. |
| **2** | Useful | Assesses whether the narrative is practically helpful for investigation, learning, escalation, or improvement. |
| **3** | Organized | Assesses whether information is presented in a logical and coherent structure. |
| **4** | Comprehensible | Assesses whether the wording is clear and understandable to a reviewer. |
| **5** | Succinct | Assesses whether the narrative is concise while preserving necessary information. |
| **6** | Synthesized | Assesses whether information from the event, investigation, and follow-up is integrated into a coherent account. |
| **7** | Internal consistency | Assesses whether information within the narrative is logically coherent and noncontradictory across the report. |
| **8** | Fair / balanced | Assesses whether the narrative is neutral, proportionate, and non-blaming. |

# Item Definitions and Scoring Anchors

## Item 1. Thorough

**What was assessed:** Assesses whether the narrative contains enough relevant information to support review of the incident.

**Table S5.** Scoring anchors for Item 1, thorough.

| **Score** | **Quality level** | **Scoring anchor** |
| --- | --- | --- |
| 5 | High quality | The narrative contains comprehensive and relevant information needed to support review of the incident, including key context, investigation findings, patient outcome or impact, and actions. |
| 4 | Good quality | The narrative contains most relevant information needed for review, with only minor omissions that do not substantially limit interpretation. |
| 3 | Moderate quality | The narrative contains some relevant information, but important details are missing, underdeveloped, or require clarification. |
| 2 | Limited quality | The narrative contains minimal relevant information and has substantial gaps that limit review of the incident. |
| 1 | Low quality | The narrative is too sparse, superficial, or incomplete to support meaningful review. |

## Item 2. Useful

**What was assessed:** Assesses whether the narrative is practically helpful for investigation, learning, escalation, or improvement.

**Table S6.** Scoring anchors for Item 2, useful.

| **Score** | **Quality level** | **Scoring anchor** |
| --- | --- | --- |
| 5 | High quality | The narrative provides actionable information that can inform review, learning, follow-up, or improvement planning. |
| 4 | Good quality | The narrative is mostly useful for review or follow-up, with minor clarification needed before action can be planned. |
| 3 | Moderate quality | The narrative is somewhat useful but requires additional interpretation or clarification before it can support action. |
| 2 | Limited quality | The narrative has limited usefulness and would require substantial follow-up before it could inform investigation, learning, or improvement. |
| 1 | Low quality | The narrative has limited practical value for investigation, learning, or improvement. |

## Item 3. Organized

**What was assessed:** Assesses whether information is presented in a logical and coherent structure.

**Table S7.** Scoring anchors for Item 3, organized.

| **Score** | **Quality level** | **Scoring anchor** |
| --- | --- | --- |
| 5 | High quality | The narrative is logically sequenced and easy to follow, with related details grouped coherently. |
| 4 | Good quality | The narrative is generally well organized, with only minor sequencing or grouping issues. |
| 3 | Moderate quality | The narrative has a recognizable structure, but some details are fragmented, repetitive, or out of sequence. |
| 2 | Limited quality | The narrative has weak organization, and the reader must work to connect key details across sections. |
| 1 | Low quality | The narrative is disorganized, difficult to follow, or presented as disconnected fragments. |

## Item 4. Comprehensible

**What was assessed:** Assesses whether the wording is clear and understandable to a reviewer.

**Table S8.** Scoring anchors for Item 4, comprehensible.

| **Score** | **Quality level** | **Scoring anchor** |
| --- | --- | --- |
| 5 | High quality | The narrative uses clear language, resolves references, and can be understood without substantial re-reading. |
| 4 | Good quality | The narrative is mostly clear, with only minor wording, abbreviation, or reference issues. |
| 3 | Moderate quality | The narrative is generally understandable, but some wording, abbreviations, or references are unclear. |
| 2 | Limited quality | The narrative is only partly understandable and contains several unclear phrases, unresolved references, or ambiguous statements. |
| 1 | Low quality | The narrative is difficult to understand because of unclear language, unresolved references, or ambiguity. |

## Item 5. Succinct

**What was assessed:** Assesses whether the narrative is concise while preserving necessary information.

**Table S9.** Scoring anchors for Item 5, succinct.

| **Score** | **Quality level** | **Scoring anchor** |
| --- | --- | --- |
| 5 | High quality | The narrative is concise and focused, with minimal unnecessary repetition while retaining important details. |
| 4 | Good quality | The narrative is mostly concise, with minor repetition or non-essential detail that does not obscure key information. |
| 3 | Moderate quality | The narrative is moderately concise but includes some repetition, excessive detail, or avoidable wording. |
| 2 | Limited quality | The narrative is frequently repetitive, overly detailed, or cluttered, making key points less easy to identify. |
| 1 | Low quality | The narrative is overly lengthy, repetitive, or cluttered, making key information difficult to identify. |

## Item 6. Synthesized

**What was assessed:** Assesses whether information from the event, investigation, and follow-up is integrated into a coherent account.

**Table S10.** Scoring anchors for Item 6, synthesized.

| **Score** | **Quality level** | **Scoring anchor** |
| --- | --- | --- |
| 5 | High quality | The narrative synthesizes relevant information into a coherent summary rather than copying or listing disconnected entries. |
| 4 | Good quality | The narrative mostly integrates relevant information into a coherent account, with minor duplication or weak linkage between details. |
| 3 | Moderate quality | The narrative partially integrates information, but some sections remain list-like, duplicated, or insufficiently connected. |
| 2 | Limited quality | The narrative shows limited synthesis and relies heavily on copied, repeated, or disconnected information. |
| 1 | Low quality | The narrative largely repeats raw entries or fragments without meaningful synthesis. |

## Item 7. Internal consistency

**What was assessed:** Assesses whether information within the narrative is logically coherent and noncontradictory across the report, including chronology, persons involved, incident mechanism, clinical findings, patient outcomes, and actions taken. This item evaluates consistency within the report rather than factual accuracy against external records.

**Table S11.** Scoring anchors for Item 7, internal consistency.

| **Score** | **Quality level** | **Scoring anchor** |
| --- | --- | --- |
| 5 | High quality | The narrative is fully coherent and internally consistent, with no material contradictions or unexplained discrepancies across its different sections or statements. |
| 4 | Good quality | The narrative is generally coherent and internally consistent, with only a minor inconsistency or ambiguity that does not affect interpretation of the incident. |
| 3 | Moderate quality | The narrative contains some noticeable inconsistencies, unclear transitions, or discrepancies, but the overall incident can still be understood with limited inference. |
| 2 | Limited quality | The narrative contains multiple or important inconsistencies that create uncertainty about key aspects of the incident, such as what occurred, when it occurred, the patient outcome, or the actions taken. |
| 1 | Low quality | The narrative contains substantial contradictions, incompatible information, or an illogical sequence, making it difficult to determine a coherent and reliable account of the incident. |

## Item 8. Fair / balanced

**What was assessed:** Assesses whether the narrative is neutral, proportionate, and non-blaming.

**Table S12.** Scoring anchors for Item 8, fair / balanced.

| **Score** | **Quality level** | **Scoring anchor** |
| --- | --- | --- |
| 5 | High quality | The narrative is balanced and factual, avoids unsupported judgments, and acknowledges relevant context or system factors where documented. |
| 4 | Good quality | The narrative is mostly balanced and factual, with only minor wording that could be more neutral or contextualized. |
| 3 | Moderate quality | The narrative is mostly factual but contains some wording that may be judgmental, one-sided, or insufficiently contextualized. |
| 2 | Limited quality | The narrative contains several judgmental, one-sided, or poorly supported statements that may affect balance. |
| 1 | Low quality | The narrative is strongly judgmental, blame-oriented, unsupported, or unbalanced. |

# Score Calculation

**Table S13.** Score calculation rules for the adapted PDQI mean.

| **Score** | **Formula** | **Interpretation** |
| --- | --- | --- |
| Item score | Each domain scored from 1 to 5 | Higher scores indicate better narrative quality for that domain. |
| Adapted PDQI mean score | Mean of the 8 domain scores | Higher mean scores indicate better overall narrative quality. |
| No separate overall item | Overall narrative quality was not separately scored in the final analytic specification | Use the 8-domain mean for report-level comparison between study periods or groups. |

# Reviewer Scoring Principles

- Rate only the completed report text provided for review; do not use group allocation, personal knowledge of the case, or external records.
- Score narrative quality rather than incident severity. A serious incident can still have a low-quality narrative, and a low-harm incident can have a high-quality narrative.
- Do not treat length alone as quality. A high-quality narrative should be sufficiently detailed, but also organized, synthesized, and concise.
- Placeholders such as missing-information should be treated as unresolved documentation gaps. They may reduce scores for thoroughness, usefulness, comprehensibility, or synthesis depending on context.
- Avoid scoring based on agreement with the recommendations. Score whether the recommendations are clearly presented, understandable, and useful within the documented narrative.
- When uncertainty remains after reading the full narrative, choose the lower score unless the criterion is clearly demonstrated.

# References

12. Stetson PD, Bakken S, Wrenn JO, Siegler EL. Assessing electronic note quality using the Physician Documentation Quality Instrument (PDQI-9). Appl Clin Inform. 2012;3(2):164-174. [doi: 10.4338/aci-2011-11-ra-0070] [Medline: 22577483]

3. Minimal information model for patient safety incident reporting and learning systems: user guide. World Health Organization; 2016. URL: https://iris.who.int/server/api/core/bitstreams/a13b925e-fe8e-49d3-a31e-7e7a7d88e646/content [Accessed 2026-07-25]
