# Supplementary material for: Nurse-Led Ambient AI Scribe for Patient Safety Incident Investigation Reports (Project NARRATE): Retrospective Pre-Post Comparative Document-Quality Study
Source: JMIR Nurs. 2026 Aug 10;9:e100775. doi: 10.2196/100775 (PMC13456676; doi:10.2196/100775)
Supplement: Supplementary file 3 [file nursing-v9-e100775-s003.docx]

Multimedia Appendix 3. SQUIRE 2.0 Reporting Checklist

Manuscript: Nurse-Led Ambient AI Scribe for Patient Safety Incident Investigation Reports (Project NARRATE): Retrospective Pre-Post Comparative Document-Quality Study

Source checklist: Ogrinc G, et al. SQUIRE 2.0 (Standards for QUality Improvement Reporting Excellence).

| **Item** | **Section** | **Checklist prompt** | **Reported** | **Location in manuscript** |
| --- | --- | --- | --- | --- |
| 1 | Title | Indicate that the manuscript concerns an initiative to improve health care. | Yes | Title |
| 2a | Abstract | Provide adequate information to aid in searching and indexing. | Yes | ABSTRACT |
| 2b | Abstract | Summarize key information from all sections of the text. | Yes | ABSTRACT |
| 3 | Problem Description | Nature and significance of the local problem. | Yes | INTRODUCTION: Background |
| 4 | Available Knowledge | Summary of what is currently known about the problem, including relevant previous studies. | Yes | INTRODUCTION: Background |
| 5 | Rationale | Informal or formal frameworks, models, concepts, or theories used to explain the problem and why the intervention was expected to work. | Yes | INTRODUCTION: Rationale for the Present Intervention |
| 6 | Specific Aims | Purpose of the project and of this report. | Yes | INTRODUCTION: Study Objectives and Hypothesis |
| 7 | Context | Contextual elements considered important at the outset of introducing the intervention(s). | Yes | METHODS: Setting |
| 8 | Intervention(s) | Description of the intervention(s) in sufficient detail that others could reproduce it. | Yes | METHODS: Intervention: NARRATE Supervisor Workflow; AI Governance and Safety Checks |
| 9 | Study of the Intervention(s) | Approach used to assess whether the intervention was associated with the observed outcomes. | Yes | METHODS: Study Design; Plain-Text Re-Rating Sensitivity Analysis |
| 10 | Measures | Measures chosen for studying processes and outcomes, validity/reliability, ongoing assessment, and contextual elements. | Yes | METHODS: Outcomes and Measurement; Reviewer Training, Blinding, and Scoring |
| 11 | Analysis | Qualitative and quantitative methods used to draw inferences from the data. | Yes | METHODS: Statistical Analysis |
| 12 | Ethical Considerations | Ethical aspects of implementing and studying the intervention(s). | Yes | METHODS: Ethical Considerations |
| 13a | Results | Initial steps of the intervention(s) and their evolution over time. | Yes | METHODS: AI Governance and Safety Checks; RESULTS: Report Sample |
| 13b | Results | Details of the process and outcome measures. | Yes | RESULTS: Interrater Reliability; Primary and Secondary Outcomes; Exploratory domain analyses; Plain-Text Re-Rating Sensitivity Analysis |
| 13c | Results | Contextual elements that interacted with the intervention(s). | Yes | RESULTS: Report Sample; DISCUSSION: Limitations |
| 13d | Results | Observed associations between outcomes, interventions, and relevant contextual elements. | Yes | RESULTS: Primary and Secondary Outcomes; domain analyses |
| 13e | Results | Unintended consequences such as unexpected benefits, problems, failures, or costs associated with the intervention(s). | Yes | DISCUSSION: Interpretation of Adapted PDQI-9 Findings; Limitations |
| 13f | Results | Details about missing data. | Yes | METHODS: Sample, Eligibility, and Selection |
| 14 | Summary | Key findings, including relevance to the rationale and specific aims. | Yes | DISCUSSION: Principal Findings |
| 15 | Interpretation | Nature of the association between the intervention(s) and the outcomes, comparison with other evidence, impact on people/systems, reasons for differences from expected outcomes, and costs/trade-offs. | Yes | DISCUSSION: Interpretation sections; Comparison With Prior Work; Implications |
| 16 | Limitations | Limits to generalizability and internal validity, including confounding, bias, and imprecision. | Yes | DISCUSSION: Limitations |
| 17 | Conclusions | Usefulness, sustainability, potential spread, and implications for practice and future study. | Yes | DISCUSSION: Conclusions; Future Work |
| 18 | Funding | Sources of funding and role of the funding organization(s), if any. | Yes | FUNDING |
